# Performance-environment mutual flow model using big data on baseball pitchers

**DOI:** 10.3389/fspor.2022.967088

**Published:** 2022-11-18

**Authors:** Yasuhiro Hashimoto, Hiroki Nakata

**Affiliations:** ^1^Faculty of Sustainable System Sciences, Osaka Metropolitan University, Osaka, Japan; ^2^Department of Health, Sport and Communication, Kobe University of Welfare, Fukusaki, Japan; ^3^Faculty of Engineering, Nara Women's University, Nara, Japan

**Keywords:** baseball, pitcher, Major League Baseball (MLB), hot hand, game flow

## Abstract

**Introduction:**

The study investigated the baseball pitching performance in terms of release speed, spin rate, and 3D coordinate data of the release point depending on the ball and strike counts.

**Methods:**

We used open data provided on the official website of Major League Baseball (MLB), which included data related to 580 pitchers who pitched in the MLB between 2015 and 2019.

**Results:**

The results show that a higher ball count corresponds to a slower release speed and decreased spin rate, and a higher strike count corresponds to a faster release speed and increased spin rate. For a higher ball count, the pitcher's release point tended to be lower and more forward, while for a higher strike count, the pitcher's release point tended to be to the left from the right pitcher's point of view. This result was more pronounced in 4-seam pitches, which consisted the largest number of pitchers. The same tendency was confirmed in other pitches such as sinker, slider, cut ball, and curve.

**Discussion:**

Our findings suggest that the ball count is associated with the pitcher's release speed, spin rate, and 3D coordinate data. From a different perspective, as the pitcher's pitching performance is associated with the ball and strike count, the ball and strike count is associated with pitching performance. With regard to the aforementioned factor, we propose a “performance-environment flow model,” indicating that a player's performance changes according to the game situation, and the game situation consequently changes the player's next performance.

## Introduction

Although game flow in sports is an interesting Research Topic for players and audience, the research is constrained because it is an abstract concept. Game flow is not well-defined, and sometimes used as psychological, emotional, and subjective experience during a game (1). In this case, flow is the mental state in which a person performing some activity is fully immersed in a feeling of energized focus. On the other hand, some studies suggest “the hot hand” as one concept of game flow (2–4). Chang (5) defines the hot hand phenomenon as context rather than mental state, and describes it as “when a previous attempt is successful, the probability that the next attempt will be successful is higher than if the previous attempt was unsuccessful.” These studies discuss whether the success or failure of one free throw is associated with the next in basketball. In baseball (6), bowling (7), golf (8), and volleyball (9), similar phenomena are being investigated. However, the hot hand phenomenon itself remains debatable because there are many uncertainties that have a considerable impact on the results. For example, the probability of success of the next free throw may change depending on the difference in assignment difficulty (10). One of these cognitions is stress. It is a well-known fact that physiological and psychological stresses affect the sports performance (11–17). Tanaka and Sekiya (18) examined the change of movement in golf putting by adding the psychological stress of receiving money as a reward. They showed that performance under this stress was reduced, and movement tended to be restricted. These studies were conducted in a laboratory and not in an actual game; the psychological stress in an actual game can change depending on the game situation (19). If a specific game situation causes constant psychological stress, it is possible that a change in performance is observed after that specific situation. For example, in the case of baseball, the batting average may change according to the ball and strike count. For a higher strike count, the batting average tends to decrease, while for a higher ball count, the batting average tends to increase (20). Hashimoto and Inomata (21) focused on the relationship between a real baseball game situation and player performance, wherein baseball pitchers felt nervous during a real baseball game, by recording their heart rate. They found a relationship between the ball count and players' heart rate. Therefore, it is probable that a specific game situation produces psychological stress in players, resulting in poor performance. However, if their perspectives are changed, the game situation changes with the performance of the players.

In this study, to clarify the detailed mechanism between the game situation and performance, we focused on the relationship between ball and strike counts and the pitching performance of baseball pitchers. The reason we focused on this relationship was the existence of short breaks in baseball. The baseball pitchers repeat similar throws during a game, and the starting pitcher often throws 100 or more pitches in one game. Moreover, the ball and strike count changes every moment depending on the pitching result. In a real baseball game there is a short break between each pitching. Thus, it is easily possible to examine the relationship between the game situation (i.e., ball and strike count) and performance (i.e., next pitching performance). Regarding the pitching motion, some studies reported underlying biomechanics on the elbow, shoulder, and trunk (22–27) and on the spin rate of the ball (28–30). Beyond the underlying biomechanics, Whiteside et al. (31) recently used 1,514,304 pitches from 2008 to 2014 Major League Baseball (MLB) pitchers, and they reported that the release point and ball speed reduced as the pitching time increased. In this study, based on big data used by Whiteside et al., we hypothesized that the pitching performance would change with the game situation. Indeed, the batting performance changed according to the count. Meyer (32) showed that the weighted on-base average (indicator for measuring the batter's attack power, calculated by adding a weight according to the expected score to the element involving base out such as four dead balls, single, double, home run) was 0.310 for the ball and strike count of “0-0,” 0.622 at “3-0,” and 0.196 at “0-2.” These data indicated that the batting performance was reduced under unfavorable ball counts for batters, which may be caused by psychological stress. We assumed that a similar phenomenon occurred in pitchers. To the best of the author's knowledge, no previous studies have examined the relationship between ball and strike counts and the pitching performance.

Based on this background information, this study used big data from PITCHf/x (Sportvision, Chicago, IL) and TrackMan (TrackMan, Inc. Stamford, CT) in MLB games to clarify these relationships. The MLB used PITCHf/x until 2015 and TrackMan after 2016 to measure data. Baseball Savant containing these data is available as open-source data on MLB, and it contains over 400,000 balls. some previous studies have already used this system (31, 33). For example, Glanzer et al. (34) investigated the relationship between variability in pitching kinematics and consistency in pitch location using PITCHf/x. They analyzed the data on 47 healthy baseball pitchers throwing 10 full-effort fastballs with 20 kinematic parameters. Whiteside et al. (33) focused on 7,600 pitches from 199 starting MLB pitchers, and they analyzed the performance variables that affected the pitching results. They found that some performance variables, including the maximum speed of the ball, consistent spatial release location, and various ball speeds, were directly related to the pitching results. In addition, PITCHf/x is widely used in research on big data in baseball (35, 36). These previous studies indicate the usefulness of big data from Baseball Savant in clarifying the pitching performance in baseball games. This study investigated release speed, spin rate, and ball release point (position axis) on each ball and strike count from Baseball Savant. Our previous study showed that the heart rate increased with the ball count, which indicates a direct relationship between the ball count and psychological stress (21). Furthermore, this finding was not related to the score and innings. Therefore, as the ball count increases, the pitcher may throw the ball into the strike zone to avoid walks, with the primary focus on accuracy. At this time, if Fitts' law is followed, it is presumed that the release speed will decrease. Fitts (37) examined the relationship between movement speed and accuracy, and proposed a standard motor control principle, “the speed-accuracy trade-off.” Therefore, with a more rapid movement, the goal accuracy reduces. We hypothesized that the release speed decreased as the ball count increased. In addition, we examined the relationship between the ball and strike count and release speed, spin rate, and release point.

## Materials and methods

The pitching data on MLB players were obtained from MLB.com (38) *via* “Baseball Savant (https://baseballsavant.mlb.com/).” We analyzed the data of 1,108 pitchers in the MLB official games from 2015 to 2019. We received ethical approval for this study after applying for the same at Kobe University of Welfare (Approval number: 20220820). We excluded the data of 22 pitchers with under throw pitches because the pitching form is clearly different from the normal overhand pitch. The under-throw pitchers release a ball in an under-arm action. The data included the pitcher's name, team, pitch type, dominant hand, ball and strike count (ball, strike, out), release point 3D coordinate data, release speed data, spin rate, and zone (ball location when it crosses the home plate from the catcher's perspective). In the 3D coordinate data, the X-axis was directed from the pitcher plate to the third base in the right-handed pitcher, and to the first base in the left-handed pitcher. The Y-axis was directed from the pitcher plate to the home base. The Z-axis was directed from the pitcher plate vertically upward. The calculated zone was one for the balls that passed through the strike zone and zero for balls that did not pass through the strike zone (Averaged value is referred to as “strike probability”).

The data obtained were calculated according to year. For the analysis of each type of pitch, using the data of the 4-seam, and sinker pitch types, pitchers who threw five balls or more in all ball counts from 0-0 to 3-2 (i.e., ball-strike counts: 0-0, 1-0, 2-0, 3-0, 1-0, 1-1, 2-1, 3-1, 0-2, 1-2, 2-2, and 3-2) were analyzed. If the same pitcher pitched for multiple years, the data in the recent year were adopted. By using the data of the change up, slider, cut fastball (hereinafter referred to as cut), curve, split finger, and knuckle curve pitch types, pitchers who threw five balls or more in all ball and strike counts, excluding the 3-0 count, were analyzed. The reason for the exclusion of the 3-0 count was a small sample size. All data were averaged according to different categories (year, pitcher, ball type, and ball and strike count) to handle an intra-individual (i.e., within-subject) factor.

Table 1 lists the number of data points for each type of pitch. For statistical analysis, data on the release speed, spin rate, zone, and 3D coordinate (X, Y, and Z axes) of the release point were first collected according to each ball and strike count. Second, the values that changed among each ball and strike count were calculated for all parameters, and they were then submitted to multivariate analysis of variance (MANOVA). In the data on the 4-seam, and sinker pitch types, ball counts (0, 1, 2, and 3), and strike counts (0, 1, and 2) were used as factors. In the data on the slider, change up, cut, curve, split finger, and knuckle curve pitch types, ball counts (0, 1, and 2) and strike counts (0, 1, and 2) were used as factors. Bonferroni *post-hoc* multiple comparisons were adjusted to identify the differences among ball and strike counts. The values were expressed as mean ± standard deviation, and the significance level was set at p < 0.05. SPSS Ver. 26 for Windows (IBM) was used for statistics.

**Table 1:** Number of data points for each type of pitch.

**Pitch. type**	***n***
4-seam	639
Sinker	293
Slider	141
Change up	139
Cut	122
Curve	19
Split finger	11
Knuckle curve	10

## Results

### Pitching variables for each type of pitch

Table 2 lists the average values of the release speed, spin rate, and 3D coordinate (X, Y, and Z axes) of the release point for each type of pitch. The fastest pitch type was the 4-seam (average: 149.92 km/h), and the slowest pitch type was the curve (average: 125.61 km/h). The pitch type with the highest spin rate was the curve (average: 2495.46 r/min), while that with the lowest spin rate was the split finger (average: 1549.58 r/min). The pitch type with the largest release point in the X-axis was the curve (average: 60.50 cm), while that with the smallest release point was the split finger (average: 54.38 cm). The pitch type with the longest release point in the Y-axis was the Change up (average: 185.36 cm), while that with the shortest release point was the knuckle curve (average: 174.09 cm). The pitch type with the highest release point in the Z-axis was the curve (average: 183.60 cm), while that with the smallest release point was the sinker (average: 178.42 cm). The pitch types with the highest and lowest strike probabilities were the 4-seam (average: 52.76%) and the split finger (average: 37.64%), respectively.

**Table 2:** Average values of the release speed, spin rate, 3D coordinate (X, Y, and Z axes) of the release point, and strike probability for each type of pitch.

	**(A) Speed (km/h)**		**(B) Spin (r/min)**		**(C) X (cm)**		**(D) Y (cm)**		**(E) Z (cm)**		**(F) Strike probability (%)**	
	**M**	**SD**	**M**	**SD**	**M**	**SD**	**M**	**SD**	**M**	**SD**	**M**	**SD**
4-seam	149.92	3.97	2263.37	150.30	55.41	21.47	181.50	12.04	179.33	13.99	52.76	4.26
Sinker	148.67	4.00	2144.33	140.73	56.63	21.19	184.34	13.35	178.42	12.71	51.19	4.87
Slider	137.72	4.30	2389.07	203.90	59.02	21.42	176.20	11.37	180.72	11.78	45.55	5.61
Change up	135.28	4.87	1773.46	240.65	58.08	23.52	185.36	14.19	179.95	12.59	41.72	7.28
Cut	142.30	4.16	2314.56	189.38	57.83	21.69	179.62	11.62	180.70	12.10	48.27	5.37
Curve	125.61	6.73	2495.46	226.75	60.50	19.87	175.70	10.14	183.60	14.70	47.41	4.96
Split finger	137.14	2.63	1549.58	115.41	54.38	21.98	175.14	9.67	181.61	10.42	37.64	5.85
Knuckle curve	130.00	6.09	2384.53	261.59	56.68	12.85	174.09	18.36	178.77	13.93	46.41	3.48
M	138.33	4.59	2164.29	191.09	57.32	20.50	179.67	12.64	180.39	12.78	46.37	5.21

M, mean; SD, standard deviation.

### MANOVA

Table 3 summarizes the primary effects of MANOVA on the release speed, spin rate, and 3D coordinate (X, Y, and Z axes) of the release point of each ball count in all pitches. Significant effects of ball count on the release speed were observed in the 4-seam, sinker, and change up pitch types (*p* < 0.01, η^2^ = 0.14; *p* < 0.01, η^2^ = 0.01; and *p* < 0.01, η^2^ = 0.09, respectively), and those of strike count were observed in all pitch types (*p* < 0.01, η^2^ = 0.72; *p* < 0.01, η^2^ = 0.73; *p* < 0.01, η^2^ = 0.30; *p* < 0.01, η^2^ = 0.32; *p* < 0.01, η^2^ = 0.26; *p* < 0.01, η^2^ = 0.65; *p* < 0.01, η^2^ = 0.59; and *p* < 0.01, η^2^ = 0.54, respectively) (Table 3A). These results indicated that the release speed decreased as the ball counts increased in the 4-seam, and sinker pitch types, while the release speed increased with strike counts, irrespective of the pitch type. In contrast, the release speed on the change up pitch type was significantly higher in 2 ball counts than in 0 and 1 ball counts (*p* < 0.01, η^2^ = 0.09).

**Table 3:** Main effects of release speed, spin rate, 3D coordinate (X, Y, and Z axes) of the release point, and strike probability by ball and strike count in MANOVAs.

**(A) Speed**	**Ball**	**F**	**df**	**η^2^**		**lambda**	**Strike**	**F**	**df**	**η^2^**		**lambda**	**Interaction**	**η^2^**
4-seam	^**^	105.35	1.73	0.14	3 < 2, 1 < 0	0.00	^**^	1677.36	1.35	0.72	0 < 1 < 2	0.00	^**^	0.04
Sinker	^**^	16.29	1.86	0.05	3 < 0, 1, 2	0.00	^**^	769.13	1.40	0.73	0 < 1 < 2	0.00	^**^	0.04
Slider		0.75	1.52	0.01		0.33	^**^	59.65	1.27	0.30	0 < 1 < 2	0.00	^**^	0.03
Change up	^**^	14.08	1.61	0.09	2 > 0, 1	0.00	^**^	63.70	1.30	0.32	0 < 1 < 2	0.00	^*^	0.02
Cut		2.07	1.78	0.02		0.22	^**^	41.61	1.37	0.26	0 < 1 < 2	0.00	^**^	0.05
Curve		0.44	1.31	0.02		0.25	^**^	32.78	1.06	0.65	0 < 1 < 2	0.00	^*^	0.17
Split finger		3.89	1.31	0.28		0.01	^**^	14.30	1.37	0.59	0, 1 < 2	0.01		0.02
Knuckle curve		0.12	1.07	0.01		0.78	^**^	10.70	1.06	0.54	0 < 1 < 2	0.03		0.20
Average				0.08						0.51				0.07
**(B) Spin**	**Ball**	**F**	**df**	η^2^		**lambda**	**Strike**	**F**	**df**	η^2^		**lambda**	**Interaction**	η^2^
4-seam	^**^	84.55	2.36	0.12	3 < 2 < 1, 0	0.00	^**^	146.65	1.69	0.19	0 < 1 < 2	0.00	^**^	0.01
Sinker	^**^	11.91	2.22	0.04	3, 2 < 1, 0	0.00	^**^	23.95	1.58	0.08	0, 1 < 2	0.00	^**^	0.02
Slider	^*^	3.93	1.99	0.03	2 < 1	0.03	^**^	13.24	1.55	0.09	0, 1 < 2	0.00		0.01
Change up	^**^	9.65	1.79	0.07	1, 2 < 0	0.00		0.42	1.44	0.00		0.56		0.01
Cut		0.75	1.82	0.01		0.37	^**^	16.84	1.53	0.12	0 < 1 < 2	0.00	^*^	0.03
Curve	^*^	3.81	1.79	0.18	n.s.	0.10	^**^	10.46	1.28	0.37	0, 1 < 2	0.01		0.12
Split finger		0.10	1.86	0.01		0.93		0.50	1.28	0.05		0.71		0.03
Knuckle curve		0.25	1.30	0.03		0.78	^**^	9.28	1.84	0.51	0 < 2	0.01		0.07
Average				0.06						0.18				0.04
**(C) X**	**Ball**	**F**	**df**	η^2^		**lambda**	**Strike**	**F**	**df**	η^2^		**lambda**	**Interaction**	η^2^
4-seam	^**^	30.99	1.98	0.05	0, 1, 2 < 3	0.00	^**^	271.80	1.41	0.30	2 < 1 < 0	0.00	^*^	0.00
Sinker	^**^	6.07	1.93	0.02	1, 2 < 3	0.00	^**^	140.63	1.48	0.33	2 < 1 < 0	0.00		0.00
Slider	^**^	16.69	1.88	0.11	0 < 1 < 2	0.00	^**^	54.03	1.47	0.28	2 < 1 < 0	0.00	^*^	0.03
Change up	^**^	15.75	1.89	0.10	0, 1 > 2	0.00		2.45	1.58	0.02		0.20		0.00
Cut	^**^	8.72	1.65	0.07	0, 1 < 2	0.00		3.09	1.33	0.03		0.00		0.01
Curve		0.80	1.30	0.04		0.29	^**^	13.25	1.13	0.42	2 < 1 < 0	0.01		0.01
Split finger		3.15	1.51	0.24		0.22		1.02	1.30	0.09		0.61		0.15
Knuckle curve		0.60	1.35	0.06		0.26		2.68	1.33	0.23		0.30		0.05
Average				0.09						0.21				0.03
**(D) Y**	**Ball**	**F**	**df**	η^2^		**lambda**	**Strike**	**F**	**df**	η^2^		**lambda**	**Interaction**	η^2^
4-seam	^**^	229.75	2.31	0.27	0 < 1 < 2 < 3	0.00	^**^	61.02	1.73	0.09	0 < 1 < 2	0.00	^**^	0.05
Sinker	^**^	61.53	2.39	0.17	0 < 1 < 2, 3	0.00	^**^	88.36	1.67	0.23	0 < 1 < 2	0.00	^**^	0.02
Slider	^*^	3.80	1.75	0.03	n.s.	0.07	^**^	21.08	1.50	0.13	0, 1 < 2	0.00	^**^	0.03
Change up	^**^	22.52	1.92	0.14	0, 1 < 2	0.00	^**^	20.22	1.61	0.13	0, 1 < 2	0.00		0.01
Cut	^**^	15.70	1.79	0.12	0, 1 < 2	0.00		0.37	1.46	0.00		0.72	^*^	0.02
Curve		3.35	1.38	0.16		0.18	^**^	13.99	1.47	0.44	0, 1 < 2	0.00		0.10
Split finger		0.61	1.37	0.06		0.28		3.44	1.98	0.26		0.09		0.07
Knuckle curve		0.58	1.44	0.06		0.54	^*^	5.49	1.39	0.38	n.s.	0.03		0.09
Average				0.13						0.21				0.05
**(E) Z**	**Ball**	**F**	**df**	η^2^		**lambda**	**Strike**	**F**	**df**	η^2^		**lambda**	**Interaction**	η^2^
4-seam	^**^	321.68	1.77	0.34	3 < 2 < 1 < 0	0.00	^**^	5.92	1.38	0.01	1 < 0	0.00	^**^	0.04
Sinker	^**^	82.03	1.78	0.22	3 < 2 < 1 < 0	0.00	^*^	5.30	1.43	0.02	2, 1 < 0	0.00	^**^	0.02
Slider	^**^	40.66	1.51	0.23	2 < 1 < 0	0.00	^**^	13.68	1.38	0.09	2 < 1, 0	0.00		0.03
Change up	^*^	3.31	1.92	0.02	n.s.	0.06	^**^	39.57	1.40	0.22	2 < 1, 0	0.00	^*^	0.02
Cut	^**^	33.35	1.55	0.22	2 < 1 < 0	0.00	^**^	12.43	1.38	0.09	2 < 1, 0	0.00	^**^	0.05
Curve		0.33	1.65	0.02		0.60		3.75	1.34	0.17		0.12		0.11
Split finger		1.00	1.64	0.09		0.56		2.20	1.39	0.18		0.24		0.08
Knuckle curve		4.01	1.10	0.31		0.19		4.98	1.42	0.36		0.12		0.08
Average				0.18						0.14				0.05
**(F) Strike probability**	**Ball**	**F**	**df**	η^2^		**lambda**	**Strike**	**F**	**df**	η^2^		**lambda**	**Interaction**	η^2^
4-seam	^**^	724.18	2.68	0.53	0 < 1 < 2 < 3	0.00	^**^	660.06	1.93	0.51	2 < 1 < 0	0.00	^**^	0.16
Sinker	^**^	738.34	2.43	0.57	0 < 1 < 2 < 3	0.00	^**^	384.29	1.89	0.40	2 < 1 < 0	0.00	^**^	0.11
Slider	^**^	124.88	1.72	0.47	0 < 1 < 2	0.00	^**^	338.60	1.80	0.71	2 < 1 < 0	0.00	^**^	0.09
Change up	^**^	113.00	1.75	0.45	0 < 1 < 2	0.00	^**^	305.59	1.76	0.69	2 < 1 < 0	0.00	^**^	0.04
Cut	^**^	90.41	1.72	0.43	0 < 1 < 2	0.00	^**^	230.25	1.89	0.66	2 < 1 < 0	0.00	^**^	0.06
Curve	^**^	15.27	1.39	0.46	0 < 1 < 2	0.00	^**^	51.18	1.54	0.74	2 < 1 < 0	0.00	^*^	0.18
Split finger	^**^	9.56	1.30	0.49	0, 1 < 2	0.03	^**^	23.02	1.58	0.70	2 < 1, 0	0.00		0.09
Knuckle curve		1.85	1.47	0.17		0.32	^**^	11.47	1.37	0.56	2 < 1, 0	0.01		0.26
Average				0.45						0.62				0.12

Significant differences among ball and strike count are shown as

* *p* < 0.05, and

** *p* < 0.01. The results of the *post-hoc* multiple comparison are shown on the right side. η^2^ is an abbreviation for partial η^2^. lambda is an abbreviation for Wilks' lambda.

Significant effects of ball count on the spin rate were observed in the 4-seam, slider, change up, and curve pitch types (*p* < 0.01, η^2^ = 0.12; *p* < 0.01, η^2^ = 0.04; *p* < 0.05, η^2^ = 0.03; *p* < 0.01, η^2^ = 0.07; and *p* < 0.05, η^2^ = 0.18, respectively), and those of strike count were observed in the 4-seam, sinker, slider, cut, curve, and knuckle curve pitch types (*p* < 0.01, η^2^ = 0.19; *p* < 0.01, η^2^ = 0.08; *p* < 0.01, η^2^ = 0.09; *p* < 0.01, η^2^ = 0.12; *p* < 0.01, η^2^ = 0.37; and *p* < 0.01, η^2^ = 0.51, respectively) (Table 3B). These results indicated that the spin rate decreased as the ball counts increased in the 4-seam, sinker, and slider pitch types, while the spin rate increased with the strike counts in the 4-seam, sinker, slider, cut, curve, and knuckle curve pitch types. In contrast, the spin rate on the change up pitch type was significantly higher in 0 ball counts than in 1 and 2 ball counts (*p* < 0.01, η^2^ = 0.07). No significant differences among the Z-axis coordinates on the curve pitch type were observed in the *post-hoc* tests.

Significant effects of the X-axis coordinate of the release point were observed in the 4-seam, sinker, slider, change up, and cut pitch types (*p* < 0.01, η^2^ = 0.05; *p* < 0.01, η^2^ = 0.02; *p* < 0.01, η^2^ = 0.11; *p* < 0.01, η^2^ = 0.10; and *p* < 0.01, η^2^ = 0.07, respectively), and those of strike counts were observed in the 4-seam, sinker, slider, and curve pitch types (*p* < 0.01, η^2^ = 0.30; *p* < 0.01, η^2^ = 0.33; *p* < 0.01, η^2^ = 0.28; and *p* < 0.01, η^2^ = 0.42, respectively) (Table 3C). These results indicated that the X-axis coordinate increased with the ball counts in the 4-seam, sinker, slider, change up, and cut pitch types, while the X-axis coordinate decreased with the strike counts in the 4-seam, sinker, slider, and curve pitch types. In contrast, the effects of the X-axis coordinate on the change up pitch type was significantly higher in 0 and 1 ball counts than in 2 ball counts (*p* < 0.01, η^2^ = 0.10).

Significant effects of the Y-axis coordinate of the release point were observed in the 4-seam, sinker, slider, change up, and cut pitch types (*p* < 0.01, η^2^ = 0.27; *p* < 0.01, η^2^ = 0.17; *p* < 0.05, η^2^ = 0.03; *p* < 0.01, η^2^ = 0.14; and *p* < 0.01, η^2^ = 0.12, respectively), and those of strike counts were observed in the 4-seam, sinker, slider, change up, curve and knuckle curve pitch types (*p* < 0.01, η^2^ = 0.09; *p* < 0.01, η^2^ = 0.23; *p* < 0.01, η^2^ = 0.13; *p* < 0.01, η^2^ = 0.13; *p* < 0.01, η^2^ = 0.44; and *p* < 0.05, η^2^ = 0.38, respectively) (Table 3D). These results indicated that the Y-axis coordinate increased with the ball counts in the 4-seam, sinker, change up, and cut pitch types, while the Y-axis coordinate decreased with the strike counts in the 4-seam, sinker, slider, change up, and curve pitch types. No significant differences among the Z-axis coordinates on the change up pitch type were observed in the *post-hoc* tests.

Significant effects of the Z-axis coordinate of the release point were observed in the 4-seam, sinker, slider, change up, and cut pitch types (*p* < 0.01, η^2^ = 0.34; *p* < 0.01, η^2^ = 0.22; *p* < 0.01, η^2^ = 0.23; *p* < 0.05, η^2^ = 0.02; and *p* < 0.01, η^2^ = 0.22, respectively), and those of strike were observed in the 4-seam, sinker, slider, change up and cut pitch types (*p* < 0.01, η^2^ = 0.01; *p* < 0.05, η^2^ = 0.02; *p* < 0.01, η^2^ = 0.09; *p* < 0.01, η^2^ = 0.22; and *p* < 0.01, η^2^ = 0.09, respectively) (Table 3E). These results indicated that the Z-axis coordinate increased with the ball counts in the 4-seam, sinker, slider, and cut pitch types, while the Z-axis coordinate decreased with the strike counts in the 4-seam, sinker, slider, change up, and cut pitch types.

Significant effects of the strike probability were observed in the 4-seam, sinker, slider, change up, cut, curve and split finger pitch types (*p* < 0.01, η^2^ = 0.53; *p* < 0.01, η^2^ = 0.57; *p* < 0.01, η^2^ = 0.47; *p* < 0.01, η^2^ = 0.45; *p* < 0.01, η^2^ = 0.43; *p* < 0.01, η^2^ = 0.46; and *p* < 0.01, η^2^ = 0.49, respectively), and those of strike counts were observed in all pitch types (*p* < 0.01, η^2^ = 0.51; *p* < 0.01, η^2^ = 0.40; *p* < 0.01, η^2^ = 0.71; *p* < 0.01, η^2^ = 0.69; *p* < 0.01, η^2^ = 0.66; *p* < 0.01, η^2^ = 0.74; *p* < 0.01, η2 = 0.70; and *p* < 0.01, η^2^ = 0.56, respectively) (Table 3F). These results indicated that the strike probability increased with the ball counts in the 4-seam, sinker, slider, change up, cut, curve and split finger pitch types, while it decreased with strike counts, irrespective of the pitch types.

### 3D coordinate relating to ball counts for 4-seam

**Figure 1:**
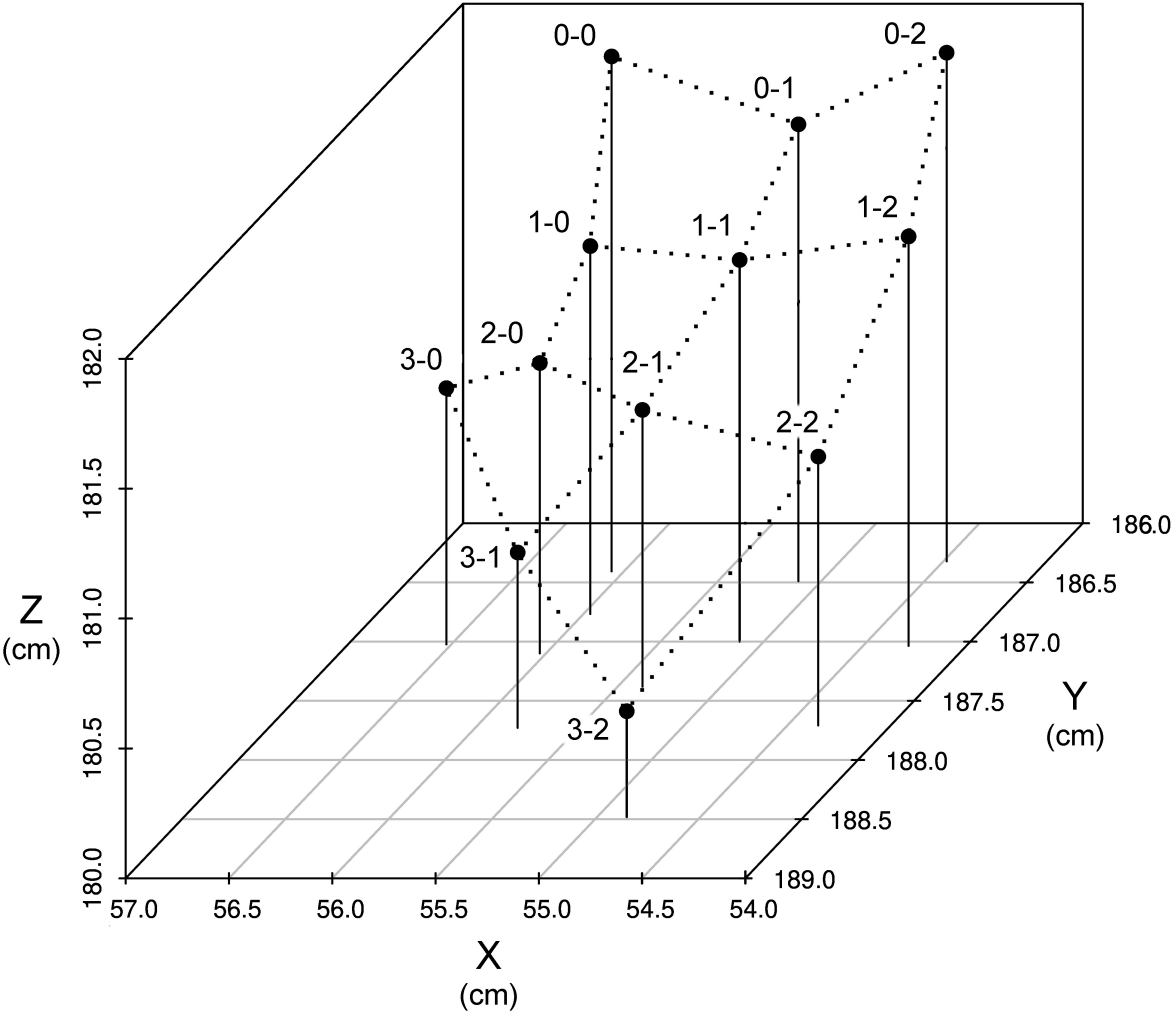
3D coordinate (X, Y, and Z axes) by ball and strike count in the 4-seam pitch type.

The averaged 3D coordinate (X, Y, and Z axes), relating to ball and strike counts for the 4-seam pitch type, is shown in Figure 1 as representative data because the number of data points was the highest among all the pitch types. From 0-0 to 3-2 count, as the ball or strike count increased, the Y-axis coordinate increased. The X-axis coordinate decreased as the strike count increased.

From 0-0 to 3-2 count, the Y-axis coordinates increased with the strike count, while the Z-axis coordinates decreased as the ball or strike count increased. The X-axis coordinates decreased as the strike count increased; however, there was no change with the increasing ball count.

Table 4 summarizes the release speed, spin rate, and strike probability for the 4-seam pitch type by ball and strike counts. Based on 0-0 counts, the release speed increased with the strike count and decreased with the ball count. The spin rate also increased with the strike count and decreased with the ball count. The strike probability decreased with the strike count and increased with the ball count.

**Table 4:** Release speed and spin rate for the 4-seam pitch type by ball and strike counts.

			**Ball count**			
			**0**	**1**	**2**	**3**
**(A) Speed (km/h)**						
Strike count	0	*M*	149.54	149.43	149.42	149.07
		*SD*	4.04	3.98	3.95	3.96
	1	*M*	150.01	149.90	149.78	149.62
		*SD*	4.04	3.98	3.98	3.91
	2	*M*	150.66	150.75	150.78	150.47
		*SD*	4.03	3.98	3.96	3.92
**(B) Spin (r/min)**						
Strike count	0	*M*	2257.55	2259.34	2256.77	2251.40
		*SD*	151.39	150.48	151.56	151.18
	1	*M*	2269.20	2265.06	2260.34	2253.15
		*SD*	149.60	152.16	152.78	150.51
	2	*M*	2275.70	2274.28	2271.81	2262.15
		*SD*	150.88	152.76	153.62	153.17
**(C) Strike probability**						
Strike count	0	*M*	54.92	56.71	57.45	62.34
		*SD*	6.27	8.39	11.89	15.60
	1	*M*	48.20	52.71	57.77	63.01
		*SD*	9.21	9.25	11.37	13.02
	2	*M*	34.86	41.27	51.48	60.71
		*SD*	12.68	10.52	10.97	10.71

M, mean; SD, standard deviation.

## Discussion

In this study, we investigated the release speed, spin rate, and ball release point (position axis) on each ball and strike count from Baseball Savant. Our main finding was that these parameters for all types of pitches were associated with the ball and strike counts.

As mentioned in the Introduction, the batting average changed according to the ball and strike count (20), suggesting that a specific game situation resulted in a change in performance. In addition, based on previous studies (21, 37), we hypothesized that there is a particular relationship between the ball and strike count and the release speed. In our data, the release speed and spin rate decreased as the ball count increased, and they increased with the strike count. It was observed from the effect size for the release speed (Table 3) that the 4-seam pitch type has the highest dependency on the ball count (η^2^ = 0.14), followed by change up (η^2^ = 0.09) and sinker (η^2^ = 0.05). However, the effect sizes of these ball types were higher in the strike count, sinker (η^2^ = 0.73), 4-seam (η^2^ = 0.72), and curve (η^2^ = 0.65). In addition, the average effect size for the release speed was higher in the strike count (η^2^ = 0.51) than in the ball count (η^2^ = 0.08). The same tendencies were observed in the data for spin rate. These results indicated a difference in the variable of effect size between the strike and ball counts, and it was greater in the strike count than in the ball count. In our findings, the pitcher would select accuracy, rather than speed, when the ball count increases, and speed, rather than accuracy, when the strike count increases. This is supported by the results of strike probability by count (Table 4C). The selection of accuracy may be related to giving a base on balls (i.e., four balls), while that for speed may be related to striking the player out. These selections indicate the relationship between ball and strike counts and the pitching performance of baseball pitchers.

In addition to the release speed and spin rate, the 3D coordinate data of the release point were associated with the ball and strike counts (Table 3 and Figure 1). Hore et al. (39) and Hore and Watts (40) examined kinematic data on the success or failure of targeting task. They reported no direct relationship between the success or failure of targeting task and the release point, and they determined that the scattered release points were interpolated by manipulating some joints of the upper limbs, including the wrist, elbow, and shoulder. However, in this study, the pitcher's release point tended to move downward and forward as the ball count increased, and toward the medial front as the strike count increased. These findings vary according to the success or failure of dart throwing and the release point, indicating specific motor control mechanisms. The term “batting-practice fastball” is used when a pitcher focuses on accuracy rather than speed. Our data for the pitcher's release point depending on ball and strike counts may indicate this term as scientific data.

Judging from our data, these phenomena were particularly significant for the 4-seam pitch type. Indeed, the number of pitchers for the sinker pitch type was the second largest, but that for the 4-seam pitch type was approximately 2.18 times the number for the sinker (Table 1).

Previous studies on “hot hand” examined the direct relationship between performances based on free throws (2–5, 10). However, these studies denied the existence of this phenomenon because there are many uncertainties affecting the results. For example, previous studies have shown that cognitive contexts change during the presentation of continuous video (41, 42). This suggests that the differences in the cognitive context of the game situation may affect the probability of successful free throws. That is, if people perceived an unsuccessful shot as the “worst failure,” the outcome of the next shot may be negative. In contrast, if the unsuccessful shot was perceived as the “next step,” the outcome may be positive. In addition, the probability of a successful free throw shot may be associated with factors such as shooting position and form. As an extreme example, a shot that did not follow a player's intended course could still hit the backboard and enter the hoop. Human behavior can be determined within a context and by certain factors, unlike coin tosses. Furthermore, the pitcher's release speed changes with the ball and strike count, suggesting that the pitcher's injury burden due to pitching also changes during the game (43, 44). This study may be extended to the discussion of the pitch limit for pitchers.

**Figure 2:**
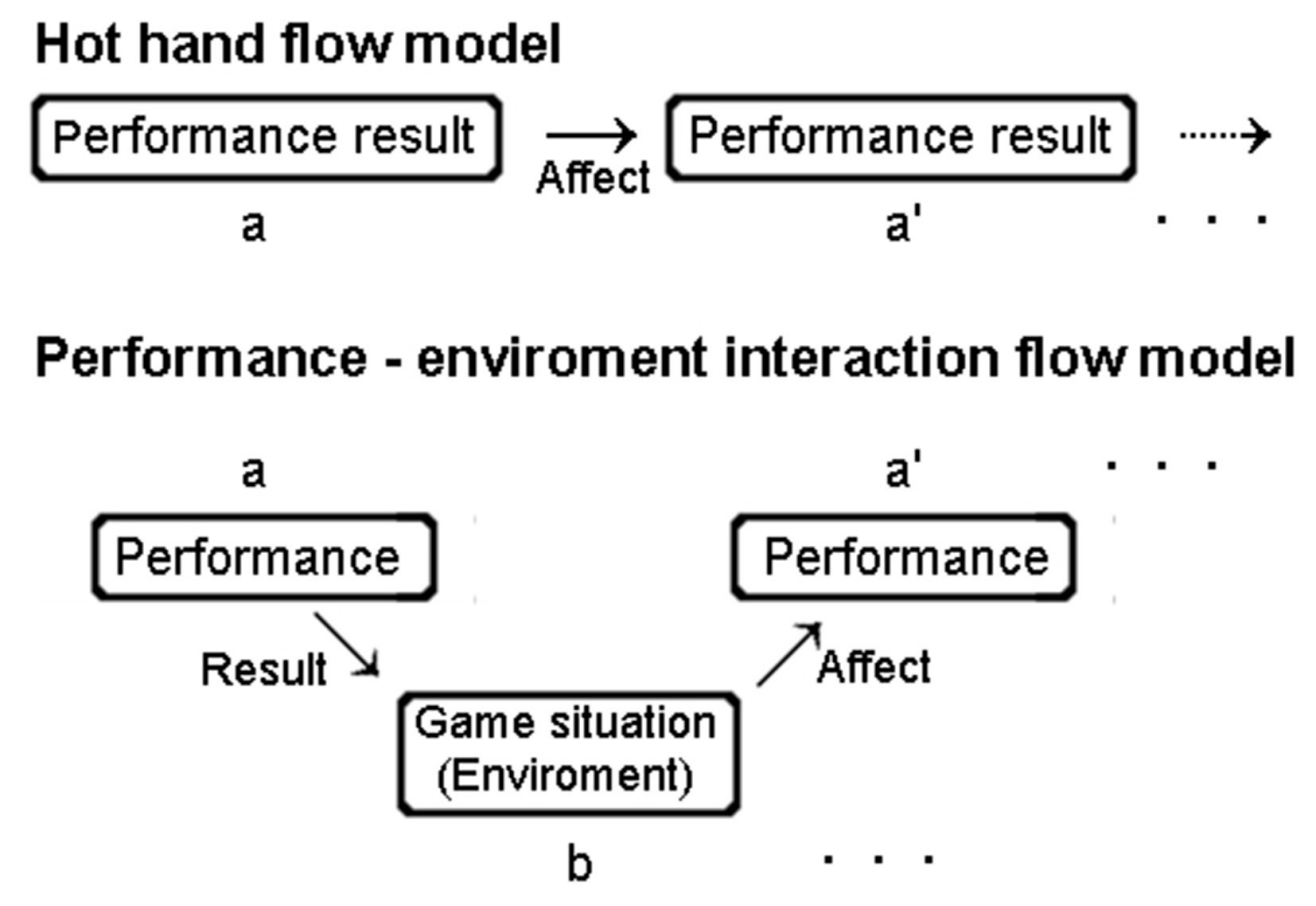
Performance-environment interaction flow model.

The findings of our study indicated that the ball and strike count was consistently associated with the pitcher's release speed, spin rate, and 3D coordinate data. From a different perspective, as the pitcher's pitching performance is associated with the ball and strike count, similarly, the ball and strike count is associated with the pitching performance. Therefore, this phenomenon could not be explained by previous models such as “hot hand.” With regard to the aforementioned factor, we propose a “performance-environment flow model,” which indicates that a player's performance changes with the game situation, and the game situation consequently changes the player's next performance (Figure 2). In sports sciences, there are models and concepts similar to our model, such as “constraints theory” (45) or a “constraints-led approach” (46). These models propose that individual, task levels, and environmental factors are related to performance and motor learning. The similarity between these and our model was the focus on the relationship between the environment and performance. However, the main difference is that our model suggested the “continuous” relationship between the environment and performance on time-series basis. In addition to this, the success or failure of a free throw in the hot hand model may be similar to that of the strike or ball in the present study. In the hot hand model, the result of the next throw (a in Figure 2) is simply considered as success or failure. However, the success or failure of the free throw is determined based on the relationship between the ball's speed and angle, and thus, it stems from a complex motion. On the other hand, our performance-environment flow model assumes the existence of a game situation (b). The next performance (a') uses a single movement index such as release speed, spin rate, and release coordinates, rather than strike/ball, which involves the uncertainty of batter's intervention. This is the primary difference between the conventional hot hand and performance-environment flow model. Currently, the versatility of our model is not examined; however, it will be studied in the future in other sports such as basketball.

This study has some limitations. First, the data reliability, accuracy, and the definition of pitch types are dependent on the PITCHf/x and TrackMan system. The origin of the 3D coordinate (X, Y, and Z axes) of the release point is presumed to be the center of the pitcher plate, and this has no clear description. In addition, the data for release speed, spin rate, and 3D coordinate were detected only at the timing of the pitcher's release of the ball. Therefore, if the kinematics data of pitching were recorded using high-speed cameras, the details of the mechanisms would be clarified. Second, the observed pitching data are based on the ball and strike counts. However, our data did not consider different game situations such as the presence or absence of runners, the position of runners, score, and inning. Third, to clarify the detailed mechanisms on the “speed–accuracy trade–off,” we need additional information about whether the next pitch was a strike or ball, and not just the ball and strike count at that time. A future study will consider these factors. Fourth, a factor of batter (ex., not-so-good batter, and high-averaged batter) is not considered to evaluate the pitching performance. These limitations should be addressed in future studies.

The findings of the study can help pitchers and coaches strengthen and understand the difference in these parameters depending on the ball and strike count. As mentioned previously, the term “batting-practice fastball” indicates that a pitcher focuses on accuracy rather than speed. Our data indicates the pitcher's release point tended to move downward and forward as the ball count increased. If pitchers could intentionally change their release point, they might be able to prevent batting-practice fastball. In other words, training methods considering the ball and strike count may improve the pitchers' performance.

## Conclusion

The present study analyzed open data published in MLB. Our findings indicate that the ball and strike count is associated with the pitcher's release speed, spin rate, and 3D coordinate data of the release point. Based on these findings, we propose a “performance-environment flow model” for baseball pitchers. This model focuses on the results of the player's performance, environment (i.e., game situation), and the player's next performance as the flow of the game, which is not a “hot hand phenomenon” that examines the relationship between the results of the player's performance.

## Data availability statement

The original contributions presented in the study are included in the article/supplementary materials, further inquiries can be directed to YH, yasu88_@hotmail.co.jp.

## Ethics statement

The studies involving human participants were reviewed and approved by Ethics Committee for Kobe University of Welfare. Written informed consent for participation was not required for this study in accordance with the national legislation and the institutional requirements.

## Author contributions

YH and HN contributed conception and design of the study. YH analyzed all data and wrote the first draft of the manuscript. HN revised the manuscript. Both authors approved the final version of manuscript.

## Funding

This study was supported by a Japan Society for the Promotion of Science KAKENHI Grant-in-Aid for Early-Career Scientists (19K24298; 20K19500) (to YH). The funders had no role in study design, data collection and analysis, decision to publish, or preparation of the manuscript.

## Conflict of interest

The authors declare that the research was conducted in the absence of any commercial or financial relationships that could be construed as a potential conflict of interest.

## Publisher's note

All claims expressed in this article are solely those of the authors and do not necessarily represent those of their affiliated organizations, or those of the publisher, the editors and the reviewers. Any product that may be evaluated in this article, or claim that may be made by its manufacturer, is not guaranteed or endorsed by the publisher.
